# MAFC: Multi-Agent Fog Computing Model for Healthcare Critical Tasks Management

**DOI:** 10.3390/s20071853

**Published:** 2020-03-27

**Authors:** Ammar Awad Mutlag, Mohd Khanapi Abd Ghani, Mazin Abed Mohammed, Mashael S. Maashi, Othman Mohd, Salama A. Mostafa, Karrar Hameed Abdulkareem, Gonçalo Marques, Isabel de la Torre Díez

**Affiliations:** 1 Biomedical Computing and Engineering Technologies (BIOCORE) Applied Research Group, Faculty of Information and Communication Technology, Universiti Teknikal Malaysia Melaka, Durian Tunggal 76100, Melaka, Malaysia; khanapi@utem.edu.my (M.K.A.G.); mothman@utem.edu.my (O.M.); 2 Ministry of education/general directorate of curricula, pure science department, Baghdad 10065, Iraq; 3 College of Computer Science and Information Technology, University of Anbar, 11, Ramadi 55431, Anbar, Iraq; 4 Software Engineering Department, College of Computer and Information Sciences, King Saud University, Riyadh 11451, Saudi Arabia; mmaashi@ksu.edu.sa; 5 Faculty of Computer Science and Information Technology, Universiti Tun Hussein Onn Malaysia, Johor 86400, Malaysia; salama@uthm.edu.my; 6 College of Agriculture, Al-Muthanna University, Samawah 66001, Iraq; khak9784@mu.edu.iq; 7 Instituto de Telecomunicações, Universidade da Beira Interior, 6201-001 Covilhã, Portugal; goncalosantosmarques@gmail.com; 8 Department of Signal Theory and Communications, University of Valladolid, 47011 Valladolid, Spain; isator@tel.uva.es

**Keywords:** fog computing, cloud computing, healthcare, multi-agent system, critical tasks management, scheduling optimization, prioritization, load balancing, resource availability

## Abstract

In healthcare applications, numerous sensors and devices produce massive amounts of data which are the focus of critical tasks. Their management at the edge of the network can be done by Fog computing implementation. However, Fog Nodes suffer from lake of resources That could limit the time needed for final outcome/analytics. Fog Nodes could perform just a small number of tasks. A difficult decision concerns which tasks will perform locally by Fog Nodes. Each node should select such tasks carefully based on the current contextual information, for example, tasks’ priority, resource load, and resource availability. We suggest in this paper a Multi-Agent Fog Computing model for healthcare critical tasks management. The main role of the multi-agent system is mapping between three decision tables to optimize scheduling the critical tasks by assigning tasks with their priority, load in the network, and network resource availability. The first step is to decide whether a critical task can be processed locally; otherwise, the second step involves the sophisticated selection of the most suitable neighbor Fog Node to allocate it. If no Fog Node is capable of processing the task throughout the network, it is then sent to the Cloud facing the highest latency. We test the proposed scheme thoroughly, demonstrating its applicability and optimality at the edge of the network using iFogSim simulator and UTeM clinic data.

## 1. Introduction

Healthcare is a fundamental part of life, where provision numerous services such as treatment, diagnosing and preventing illnesses, diseases, injuries, and mental trouble. Technology and healthcare have a very long interaction history. Many healthcare applications are developed with the help of technology to enhance our life. Healthcare applications are the most critical tasks as they directly infect patient life [1,2]. Fog computing is one of the key technologies that can be implemented in healthcare applications. Fog computing is a distributed front-end computing system that performs on the edge of the networks that ensure low latency, execution time, scalability, privacy, security, and energy consumption which they are all crucial in healthcare applications [3].

One of the crucial strategies is providing a multi-processing technology, that can be provided by Fog computing [4], to fulfill healthcare applications needs. In order to implement multi-processing technology in the Fog computing model, software Agents must be created and loaded onto the wireless sensor network Nodes [5]. Multi-Agent Systems (MAS) gained considerable attention from academics in various disciplines, including informatics and civil engineering, as a means of solving complex problems by subdividing them into smaller tasks. The individual roles are delegated to autonomous individuals, called Agents. That agents use multiple inputs to decide on a proper action to solve the problem, e.g., history of actions, interactions with neighboring Agents, and target. MAS have found many applications, including complex modeling systems, smart grids and computer networks [6]. Another study proposes a multi-agent scheme for distributed offloading computation in mobile Fogs [7]. The authors of [8] present Agent-based self-organizing model for detection rapid biological threat, that support monitoring large-scale environments efficiently and accurate biological threats detection. An Open IoT Fog Agent has been developed in [9] in which the agent provides monitoring, connection detection, autonomously takes actions, and handles the connection between Fog Node’s devices and the container.

A recent interview with emergency doctors confirmed that dividing priority into two different aspects was crucial: time-based urgency and performance-based criticality to perform the task [10]. Delivering healthcare services is a critical role [11]. Cloud services are only used when required, reducing costs of utility computing and security issues while maintaining local resources when critical processes need them [12]. In time-critical services such as healthcare, Fog computing is perceived to be cost-effective compared to Cloud computing because of its lower latency and, in some cases, because of the spare capacity of locally available resources [13]. At the edge of the network (Fog Node (FN)), resources should be available to process the most critical tasks locally and forward normal tasks at the center of the network (Cloud) [14]. 

**Figure 1:**
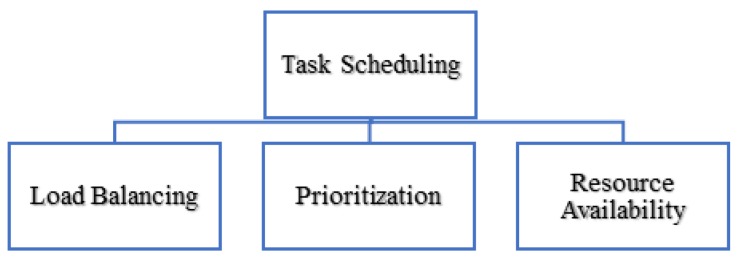
Critical task management.

Many researchers have focused only on resource management for critical tasks applications, such as the authors in [15], who have categorized all the terms resource scheduling and resource provisioning comes under the resource management. Whereas in [16] the authors mentioned that the first and most critical issue is the design of resource management techniques to schedule which analytics application modules are deployed to each edge device to reduce latency and optimize the performance. They focused on the tasks but from the view of resources. All the terms load balancing, prioritization, and resource availability significantly contribute to enhancing the performance of task scheduling (Figure 1).

Inefficient scheduling strategies face the challenges of overused and underused (imbalanced) resources leading either to loss of service efficiency (in the case of overused) or to resource wastage (in the case of underused) [15,17,18].

Load balancing is among the most critical problems to implement multi-resource scheduling tasks in a heterogeneous computing environment. If the load balancing approach is assisted in the schedule, it can influence energy consumption. The use of the CPU is also mandatory for both Service Level Agreement (SLA) and energy consumption, which directly affects the scheduling of load balancing tasks [19]. The CPU usage affects task processing and each node has its own CPU to process the tasks. Therefore, the proposed method aims to enhance task scheduling to improve task processing. 

Creating a balance of concern between resources and tasks will significantly improve the response of tasks processing and resource management. Load balancing is an essential technique in resource management that can be embedded with task management factors to get a robust system [20]. Scheduling involves finding an optimized mapping for allocating n tasks to m processors. Moreover, there are several crucial variables to enhance scheduling in Cloud computing. According to [19], the following parameters and instructions for the optimization of Cloud computing architectures are presented.

- CPU utilization: keeps the CPU as busy as possible.
- Throughput: the number of processes that complete their execution per time unit.
- Turnaround time: the amount of time to execute a particular process.
- Waiting time: the amount of time a process has been waiting in the ready queue.
- Response time: the amount of time it takes from a request was submitted until the first response produced.

For supporting healthcare applications, Personal Agents (PAs) and Fog Nodes (FNs) should fulfill a set of responsibilities. Healthcare application tasks are produced, mayhap, at high critical levels, and should be processed promptly. Resource management can be achieved by scheduling, load balancing, and resource sharing among a group of PAs and FNs. The resource management is, then, a relevant strategy that ensures the response to each healthcare application task to an appropriate and suitable resource. Scheduling, in general, is the process of sequencing the execution of a group of incoming tasks into predefined resources regardless of the availability of resources and the criticality of tasks. 

In this paper, a Fog computing model MAS based for managing critical healthcare application tasks was build that significantly manages Fog computing resources by providing two levels of task prioritization (locally and globally), scheduling, load balancing, and resource sharing through the involvement of MAS. The main contributions of the proposed model are the following: First, efficient prioritization for abnormal tasks. Second, efficient Tasks Scheduling for patient critical situations. Third, balanced network workload to local and global levels by estimating the cost of local and global workload. Lastly, facilitate node cooperation and resource sharing with neighbor Nodes by using Multi-Agents System.

The structure of this paper is as follows: Section 2 is the related work. Section 3 discussed the Research methodology. Section 4 discussed the materials and methods. Section 5 shows the modeling of Multi-Agent Fog Computing (MAFC). Section 6 describes the optimization of scheduling. Section 7 describes the role of multi-Agents. Furthermore, Section 8 is the results and discussion. Section 9 discussed the conclusion.

## 2. Related Works

Task scheduling is an essential research subject in multiple domains. It has a significant impact on edge network, and FNs as devices at the edge of the network and the computational capabilities of FNs are limited while different energy constraints are restricted. Hence, Nodes should therefore carefully select the tasks they will perform locally, making it imperative to apply smart techniques to optimize scheduling tasks. In any case, Nodes will take into account the specific characteristics of the tasks in conjunction with their existing statues. The characteristics of tasks are priority, resource load balancing, and resource availability. These characteristics significantly affect task scheduling optimization. MAS contributes to scheduling optimization by enhancing priority detection, load balancing, and resource availability. A MAS is implemented in [21], for the cooperation between different components of the energy management system using multiple Agents for smart communities, UESSs based on Fog are developed for energy management by scheduling home appliances to reduce the cost of global power and computing and to overcome the problem when energy storage is more than future demand, which can degrade the system. 

A novel approach was proposed in [22] based on a consensus algorithm to improve processing resources of the edge devices by distribution of tasks among a cluster of new edge computing aspect for dynamic distributed networks (Dew) Nodes. Whereas a job scheduling using a scheduling algorithm called RT-SANE (Real-Time Security Aware scheduling on the Network Edge) has been proposed in [23] to ensure a real-time performance in Fog network, an orchestration agent resides on each computing system in the distributed architecture. These Agents create instances that are specific to each job across the various Nodes used in the system. In load balancing, a Fog-to-Fog (F2F) data caching and selection method was proposed in [24] using a multi-agent cooperation framework to overcome resource selection and allocation problem. Conversely, [25] proposed a biologically inspired design uses a strictly distributed multi-agent method to provide self-organizing and self-healing abilities, based on embryonic development characteristics and biological cell communication to overcome the problem of IoE/ Fog operating within harsh environments, need for solutions which allow highly resilient service delivery. This multi-agent approach uses local-only communication to allow high levels of a node churn to implement a distributed processing capability. A tracking of load changes was tackled by [26], in which they proposed a distributed multi-agent based framework, using first-order consensus algorithm, structured on three-layer Fog computing design for effective maximum economic micro-grid dispatch. This system tracks shifts in load at any time of day, taking into account unexpected entries and exits of the units. In the same context, a framework that carries out weighted simplex strategy based on supervised safety control, resource management and autonomous robots confidence estimate is proposed by [27], to enhance safety infringements while showing greater optimized speed during indoor driving in which a resource manager discharges tasks to a free FN. The utilization of the resources was proposed by [28] to provide a service replication program that increases the quality of resources. The system uses a two-threshold adaptive sensing method to classify services which are expected to be repeated in the immediate future and an optimal task assignment scheme based on multi-Agents that allows for batch-wise decision taking. The QoS-aware policy should focus more on enhancing processing time on a priority basis instead of keeping all tasks [29]. The completely decentralized method is followed in most works, but consideration of the computation depends on the priority load factor is ignored [30]. Priority scheduling of tasks achieves the best result among all other schedulers [31]. Calculate a priority for every task taking into consideration the agent’s physical and emotional state and the agent’s attitude to the task and time. Tasks are then graded and the one with the top priority is chosen. Then while a task is being processed, priorities are recalculated at any time stage (tick) so that a task which has acquired a higher priority may interrupt the task if the latter is interruptible [32]. While the task is being processed in real time, the priority can be determined by the urgency of the task and its importance. Interactive message takes precedence over simple informative messages [33].

From the above, we can conclude two main things: First, prioritization of the critical tasks will contribute significantly to accurate scheduling, in which priority scheduling can achieve the best result, among other scheduling procedures. Second, from the literature, we can observe that no one of the researchers has used or implement priority factors with MAS to enhance scheduling or speed-up critical task processing. Moreover, it is essential to mention the contribution of prioritization through MASs.

## 3. Research Methodology

**Figure 2:**
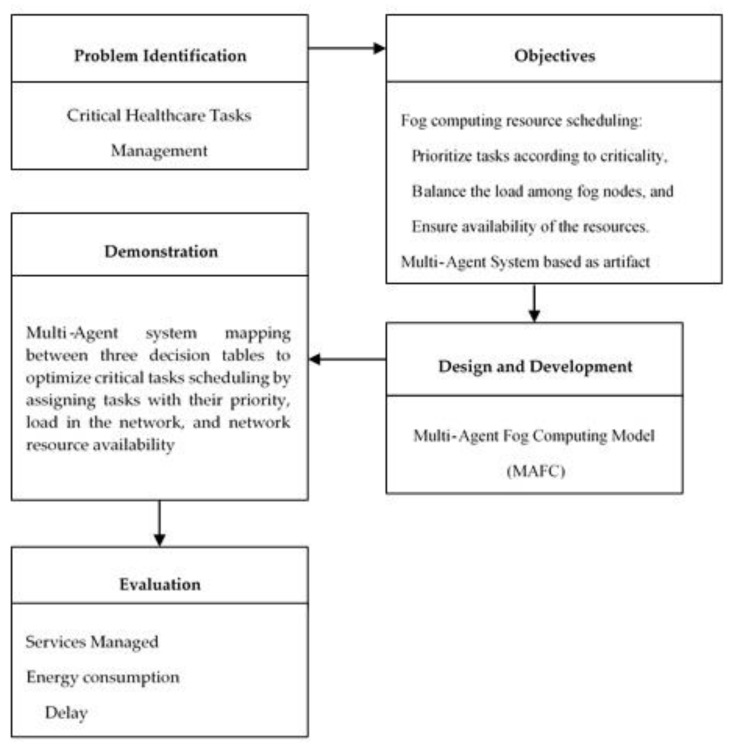
Research methodology overview.

The aim of this research is to develop an efficient resource management framework to support critical healthcare application tasks requirements. Therefore, as in any study it requires a research outline or design which serves as a working plan and guarantees that proof assembled enables the assurance of the research [34,35]. The research methodology overview is depicted in Figure 2.

In the preliminary stage, we investigate the recent studies to highlight the most crucial problem and specify the effected factors in order to find a solution. Defining the objectives of a solution and what would a better artifact accomplish in the second stage. The third stage is the designing and development of the proposed model using the artifact of previous stage. In the demonstration stage, the role of artifact to solve the problem has been identified. In the last stage, evaluation of the results by measuring services managed, energy consumption, and delay is conducted. 

### Dataset Used

The used dataset is a real dataset of human vital signs. The real dataset is adopted from UTeM clinic and covers blood pressure data and critical patient calculations. We adopt from these data the systolic, diastolic, and plus rate. In fact, the indication of a critical situation is characterized by a low or high rate of systolic, diastolic. The recorded data of the patients contains numbers only. Table 1 demonstrates the description of dataset. UTeM clinic dataset are private dataset and prohibited from publishing online due to the rules of the university to protect patients’ privacy.

## 4. Materials and Methods

For carrying the simulations, iFogSim [16] has been used. This simulator permitted the modeling of different functions in sensors, PAs, FN, and Cloud levels. The iFogSim simulator is capable of testing various scheduling techniques for Fog and Cloud conditions, hence it was extremely useful. This simulator goes during the sensor → PA → FN → Cloud. Therefore, it becomes pertinent for various devices that are edge-enabled. In the MAFC model, there are two task priority queues. The first queue is at PAs’ level, which is considered as local prioritization due to each PA will prioritize its local connected sensor tasks. The second queue will be at the FN level, and this queue will be done by Fog node Agents (FNAs), which is considered as global prioritization due to re-prioritizing the tasks among all connected PAs. Three arrays have been created to represents the priority decision table (PDT), Resource Availability Decision Table (RADT), and Load Balancing Decision Table (LBDT). The FNAs at FN level will match among decision tables in order to select the tasks with the highest priority and assign the suitable available resources to process with a balanced load.

## 5. Modeling of MAFC Healthcare Resource Management Optimization

The model contributes to responding to an abnormal patient’s situation. Assigning priority to each patient will significantly improve their treatment, by reducing the time of responding, and also saving the patient’s life in a highly critical situation. On the other hand, managing network resources will reduce the cost of establishing enormous resources, in which all the resources can be balanced by assigning task priority scheduling procedure. The MAFC model consists of three main tiers: Cloud tier, Fog tier, and user tier. The Agents will take the responsibility of communication between the tiers. Handling patients’ sensor signals will be done by a group of PAs. Each group of patients’ sensors is connected to one PA. The primary duty of PA is to prioritize the incoming signals by identifying critical and normal tasks. Normal tasks will be forwarded to Cloud tier, and abnormal tasks will be forwarded immediately to Fog node agent (FNA).

We consider a set of patients, i.e., S = {s1, s2, …, sn} each sensor represents a patient, responsible for sending the sensed data continuously. Each group of sensors is connected to one Personal Agent (PA). On top of PAs, a set of FNs, i.e., N = {n1, n2, …, nn} execute a set of tasks for processing. A task-flow T is resorted two times, according to the priority. First prioritization is done locally by PA, in which the PA will sort all the incoming tasks from connected sensors. Second, the prioritization is made by the FNA, in which reprioritization occurs globally among all the incoming tasks from PAs. The FNA will receive a series of ordered tuples ‹L, T, R›, where L is the load at local node and global Nodes, and R is the availability of the resources. The processing of each task should save the patient’s life by ensuring the fastest response, such as notifying the nearest available doctor in the hospital or informing the patient’s relatives. Every task is accompanied by its priority, which is measured according to the level of patient criticality situation. A four-tiered model architecture is proposed in this paper based on priority mechanism in the Agents as a complementary method of task scheduling optimization in two levels locally and globally.

**Figure 3:**
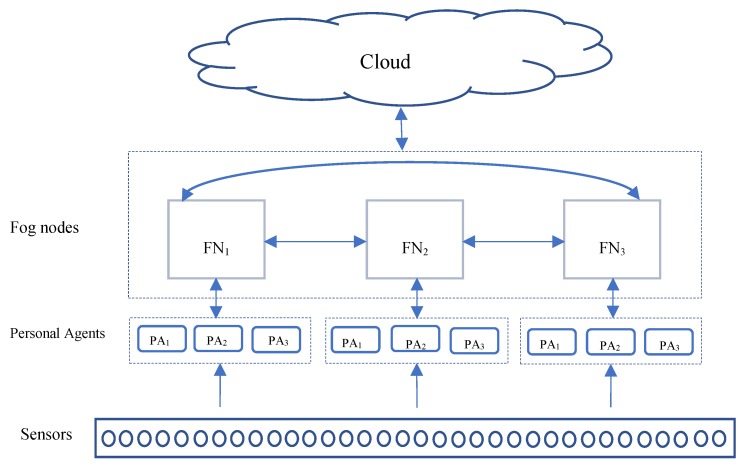
The flow of tasks from sensors to Cloud.

In Figure 3, four-level model architecture is shown, in which levels are placed in two types of network sensors, and PAs are located in local area networks, whereas FNs are located in the intermediate layer and Cloud in the global area network. 

All the sensed tasks by sensors will be filtered by their local PA, in which normal tasks will be forwarded to Cloud immediately, and the abnormal tasks will be transmitted to the connected FN.

The most critical problem is the distribution nature of the task scheduling optimization, which will lead to consume time and resources. It will further exhaust and disturb the work of the network. The proposed model solves this problem by using multi-agent technology, which can make local decisions by individual agent and distribute decisions of MAS.

In the above figure, each group of sensors is connected to three PAs, and each three of them is connected to one FN. After receiving task T from PA, FN N should take one of three actions: either execute T locally, or send T to nearest available FN, or send T to Cloud. The actions mentioned above will be taken by a multi-agent system, in which a MAS will decide each task should be processed in which place.

**Figure 4:**
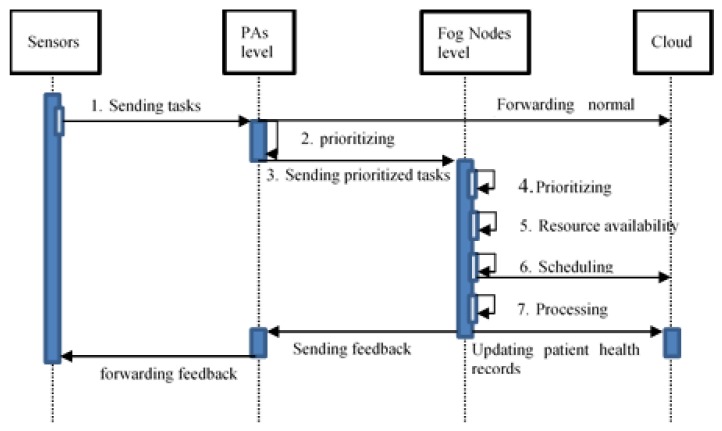
Sequence diagram illustrating the interaction protocol.

A protocol for interaction between these components is proposed based on the components of the MAFC model architecture above. There are three different cases: (a) local FN executes the task, (b) neighboring FNs execute the task, and (c) Cloud is responsible for accomplishing the task. Figure 3 provides a sequence diagram for those three different cases. A sequence diagram for these three different cases is provided in Figure 4. The goal here is to present a protocol of best efforts to handle the incoming tasks without failures, and task loses. If there is no connection to the Cloud for any reason, for example disaster, the task will remain on the waiting list, concerning priority, until a resource is available to process it.

MAFC model’s decision relates to defining and selecting the correct node to process any ’rejected’ mission. We rely on the multi-attribute utility theory [36], Where we suggest that the above mentioned tuples are characteristic of each peer ‹L, T, R›, where L is the load at local node and global Nodes and R is the availability of the resources. Since each node is defined by multiple attributes, we strive to ’combine’ them and provide a final ranking in order to select the most suitable node. Multi-attribute utility theory concerns communicating multiple attribute utilities (called as individual utilities) as a function of each attribute’s utilities taken individually.

In the first case, a PA delivers a task to local FN. We believe FN complies with all required requirements of resource availability and load balancing for the submitted task. The norm is that this task is processed at local FN. In the second case, The PA submits a task that cannot be carried out on a local FN with the execution of other tasks, or local FN does not have enough resources to execute the task. The scenario, for example, in which local FN is busy, the critical task cannot wait in a queue.

In such cases, local FNA interacts with neighbor FNAs to find the suitable resources and forward the task to be processed. This is just one example of a scenario in which load is balanced according to resource availability in the FN level.

In the third case, when all FNs are busy, where the load is balanced among FNs, but there is no resource availability. Then, the task will be forwarded to the Cloud in order to be processed.

The complexity of a task will be calculated depending on task completion time, consumed resources, and available resources. Therefore, for the tasks that are more complex, agents will reserve more nearest resources to ensure a fast response. 

Priority and complexity parameters depict two crucial aspects of a task execution process, i.e., an indication of the immediate initiation of its execution (priority) and of the time, resources availability, and load balancing required for execution (complexity).

### Motivating Scenario

**Figure 5:**
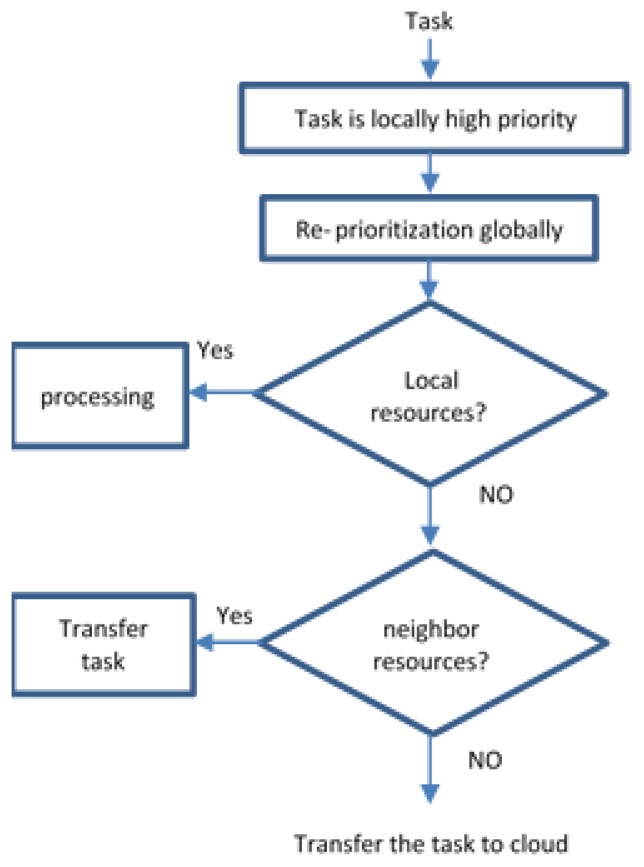
Internal process of each task in Personal Agent (PA) and Fog node agents (FNAs).

Assume that we want to monitor a group of patients in a hospital for detecting emergency situations such as blood pressure (PB). A group of PAs are inside the hospital and are responsible for collecting and evaluating the patient’s sensed data. PAs are characterized to evaluate the criticality of each patient and sorting the abnormal signals from high critical first to low critical at last. Each PA will forward the abnormal tasks with high priority to the FNA. Nodes have specific resources and can process small amounts of data. Each group of sensors is connected to one PA, and each group of PAs is connected to one FN. After each task has been issued, each node checks its resources, its load and the properties of the task and decides whether it will be executed locally. If not, the FNA chooses the most suitable neighboring node to assign the task for execution. Otherwise, the FNA sends the task to the Cloud and wait until the final response faces increased latency affecting the final response time, see Figure 5. Nodes share their load, remaining resources and speed to give the neighboring Nodes a view of their status. Sensing blood pressure continuously by sensors will produce a huge amount of data, and these data need to be evaluated along with the historical patient situation. Evaluating the data manually by the doctors will take time, which may affect the patient’s life. The procedure in our model will reduce processing time to get fast responses and notify the doctors about the patient’s situation.

## 6. Tasks Scheduling Optimization

Task scheduling in a distributed system involves allocating resources to tasks in a given order [37]. Job scheduling in Fog computing means distributing a set of tasks to FNs at the edge of the network. Applications in the Fog computing environment between the sensor and the Cloud operate on Fog devices like a gateway, switches. These resources are widespread and variable. Consequently, efficient resource scheduling is necessary to optimize the use of these resources and to increase benefit providers of Fog and Cloud [38].

The technology of software Agents has been utilized in several systems to improve the performance and quality of their services. The main tasks of Agents in Fog computing are the deployment, scheduling, and support of service codes in various FNs, in addition to the management, control, monitoring, and scheduling of available resources on a single Fog or a collection of similar heterogeneous distributed Fogs.

Each FN should apply tasks scheduling optimization scheme for the incoming tasks. In the proposed scheme, scheduling optimization depends on three parameters priority, load balancing, and resource availability ‹P, L, R›.

### 6.1. Prioritization

The completely decentralized solution is followed in most works, but consideration of the priorities and device constraints is ignored [30]. Scheduling based prioritization of tasks achieves the best result among all other schedulers [31]. Assigning a priority to a task, it is the process of ranking the task and choosing the one with the highest priority. While the task is processed in real time, the urgency and significance of the task will dictate the priority. The high-priority patients must, at every moment, be satisfied before the lower-priority ones.

**Figure 6:**
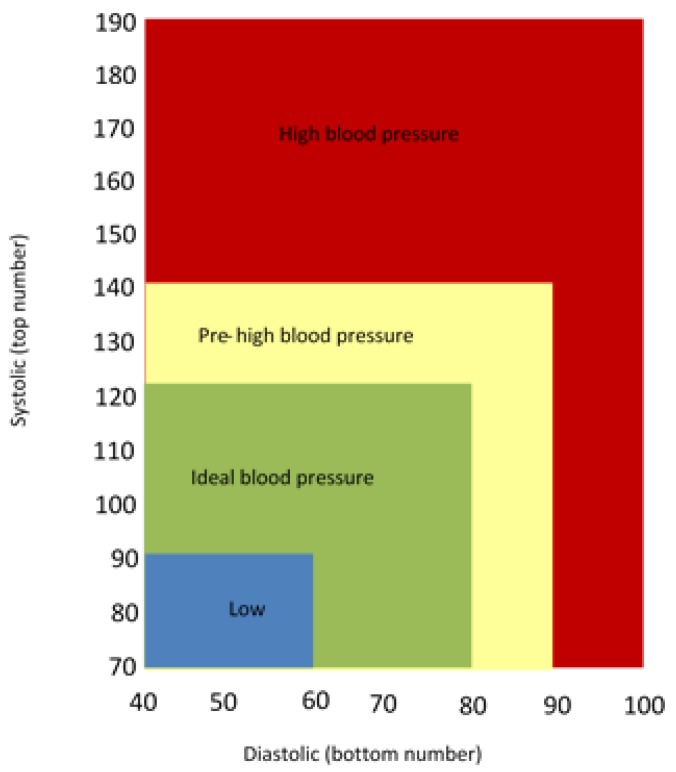
Blood pressure ranges.

Prioritization will be applied two times, first at the PA level. As mentioned earlier, each group of sensors are connected to one PA, ranking all the incoming tasks from the sensors according to the criticality of the patients will be done by PA. Critical tasks (C) list and normal tasks (N) list is the output from PAs. Algorithm 1 shows the first level of priority.
**Algorithm 1:** Forming a Local Task List Based on Priorities in PA1. **Input**: sensed tasks T from connected sensors 2: **Output:** list of sorted critical C, Moderate M, and normal N data 3:  if T = C then do, update priority list (P) else, 4:    if T = M then do,       set T as M else, 5:    set T as N 6: **end if**;

Calculating the criticality of task is based on the patient situation, in which if the sensor is measuring blood pressure, then measurement has four ranges, two of them are critical (Low and High), one is normal (Ideal), and one is pre-critical (pre-high), as shown in Figure 6.

The second level of prioritization will be in the FN among all connected PAs. Resorting the incoming tasks according to priority and creating a PDT, which consists of sorted abnormal tasks that require a fast response. Algorithm 2 represents the forming of PDT.
**Algorithm 2:** Forming a PDT Based on Priorities FNA**Require (input):** algorithm 1 // criticality C of every task in PA
**Ensure (output):** PDT 1. The C of T is calculated for all PAs // Re-prioritization globally among all PA 2. The normal (N) of T is calculated for all PA 3. Empty Priority list (P) and stack (S) 4. Push the C into stack S in decreasing order 5. While the stack S is not empty do 6. If top (S) is not the highest critical C then 7. S ← the highest critical C 8. Else 9. P ← top (S) 10. pop top (S) 11. End if 12. End while

In order to model the priority aspects, each task t is assigned to priority level. If the task is critical C, the priority will be high (HP), whereas, if the task is moderate M, the priority level is moderate (MP), and if the task is normal, then the priority level is low (LP).

### 6.2. Load Balancing

Considering a situation where a FN accepts a request for data processing, processing the request and responding. However, if the FN is busy handling other requests, it can only process part of the payload and discharge the remaining parts to other FNs. There are two approaches to patterning interactions between FNs. Firstly, the centralized model, which uses a central node to control the offload interaction between the FNs. Secondly, where each FN executes a protocol to distribute its current state information to neighboring Nodes. Every FN then keeps a dynamically modified list of the best Nodes that can support the tasks offloading [39]. In Fog computing environment, the offloading method can be done through multi-agent due to its distributed method, especially in load balancing issue Reducing the time required to determine the final allocation and ensuring the load balancing to maximize performance.

### 6.3. Resource Availability

Resource availability is particularly relevant in dynamic life-critical environments such as healthcare, which involves many circumstances in which there are numerous tasks and limited resources to handle all tasks. Furthermore, resource availability is a crucial factor that significantly affects the processing of critical tasks.

The Fog layer contains multiple FNs. Each FN checks its local resource availability that required for critical task execution. Further, if the local resources are unavailable, the request can be forwarded to the nearest neighbor Nodes or Cloud layer for execution in case of unavailability of resources at the FNs level in the worst case. 

**Figure 7:**
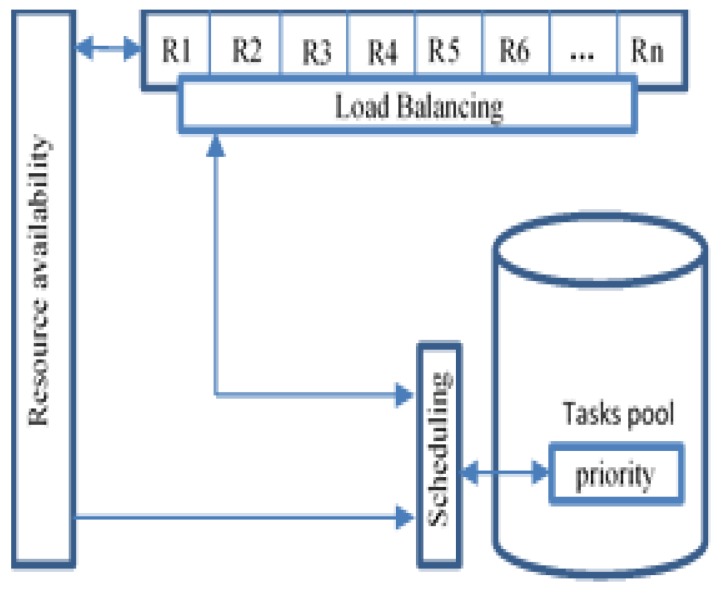
Scheduling optimization supporting.

Figure 7 shows the contribution of priority, load balancing, and resource availability to optimize critical task scheduling to ensure a fast response.

## 7. MAS Role in MAFC Model

Handling patients’ sensor signals will be done by a group of PAs. Each group of patients’ sensors is connected to one PA. The primary duty of PA is to prioritize the incoming signals by identifying critical and normal tasks. Normal tasks will be forwarded to Cloud tier, and abnormal tasks will be forwarded immediately to the FNA. 

**Figure 8:**
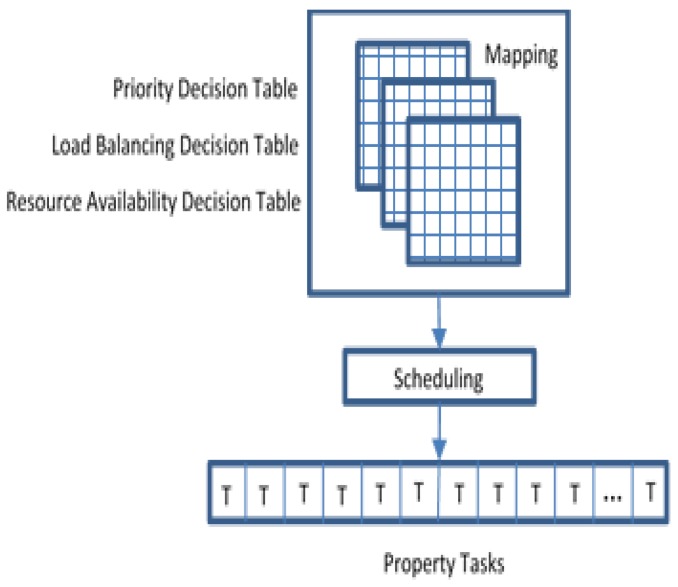
Multi-Agent System (MAS) role in Multi-Agent Fog Computing (MAFC) model.

As mentioned earlier, three main parameters support task scheduling optimization. Each one of these parameters will have a decision table that contains the basic information. The PDT consists of sorted abnormal tasks that required fast response. The LBDT consists of the current load of each resource, local and global, in which all the resources may have balanced load, but they do not have resource availability. RADT consists of suitable available resources among all network resources, see Figure 8.

The primary role of MAS is mapping between these three decision tables to optimize scheduling the critical tasks by assigning tasks with their priority, load in the network, and network resource availability. Tasks scheduling will schedule the tasks significantly by assigning tasks property that consists of when and where each task should be processed. When a FN suddenly became slow or busy, the FNA will transfer all the tasks to the nearest available FN. Algorithm 3 is a modified version of previous algorithms presented in [40,41].
**Algorithm 3: Scheduling-Based PDT, RADT, & LBDT****Input:** PDT **Output:** Property Tasks 1. // call algorithm 2 to form the list of tasks based on priorities 2. OperatingSystem.getLocalNodeAvailableProcessors()/ 3. OperatingSystem.getLocalNodeLoadAverage()/ 4. OperatingSystem.getGlobalNodeAvailableProcessors()/ 5.OperatingSystem.getGlobalNodeLoadAverage()/ 6. OperatingSystem.getCloudAvailableProcessors()/ 7. Map < Integer, Map < String, Double>> ResourceAvailableTable = new //RADT is created 8. Map < Integer, Map < String, Double>> LoadBalanceTable = new //LBDT is created 9. from PDT, RADT, and LBDT do   Select the best match then,   Forward to scheduling then,   Property Tasks List (PTL) is created   Deploy PTL   Deployed = true 10. If Deployed then 11. return successful

## 8. Experimental Evaluation

In this experimental evaluation, we analyze whether the MAFC model can process the critical tasks locally or transfer to the appropriate resources. To illustrate the feasibility of our proposed MAFC model and interoperation with Cloud-based solution, we follow the Java-created simulator (iFogSim) toolkit to simulate both the environment and the integrated architecture. We conduct the simulation in three phases. At first, MAFC’s performance is compared with the Cloud-based solution in terms of managed services, energy consumption and delay are sufficient in Fog, considering computational resources. For modeling, synthetic workload is not currently available as the real-world workload for simulating such a large-scale environment. The real dataset used in this simulator is adopted from the UTeM clinic and covers blood pressure data and critical patient calculations. the dataset composes of a measurements range for 6740 records. In fact, the indication of a critical situation is characterized by a low or high rate of systolic, diastolic, see Table 2. In the scenario of this paper, the decision to perform a task locally is assisted by a high priority, low load, and high resource availability.

### 8.1. Services Managed

The purpose of services managed is to present the benefit of using Fog devices to increase Cloud data centers capability. On the one hand, FNs are responsible for processing the critical tasks. On the other hand, cloud datacenters are responsible for executing normal tasks and critical (in case of unavailability of FNs resources). Increasing or decreasing the number of tasks depends on the real dataset. The performance was assessed considering the number of services managed, the number of Cloud datacenters and the number of Fog Nodes. When the number of Cloud datacenters is two and Fog Nodes is four, the number of tasks that was processed was 2546.2 in FNs, and 1797 tasks only have been processed in Cloud datacenters. Alternatively, when the number of Cloud datacenters was three and the same number of FNs, the results are 8213.6 in Fog Nodes and 2590 in Cloud datacenters. Then the number of Cloud datacenters has been fixed to 4, and the number of FNs is 4. The results were 10,227.4 tasks processed in FNs and 3934 tasks in Cloud datacenters. With the same number of Cloud datacenters and 8 FNs, 12,306.6 tasks have been processed in FNs and 5908 tasks in Cloud datacenters. Furthermore, when the number of FNs was increased to 12, 13,364 tasks have been processed in the FNs and 9883 tasks processed in Cloud datacenters. Lastly, when FNs increased to 16, 3894, tasks are processed in FNs, and 1257 tasks are processed in Cloud datacenters.

**Figure 9:**
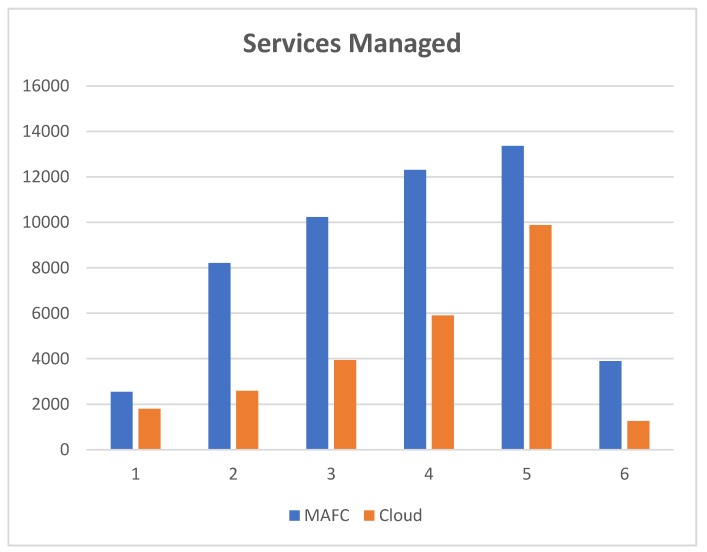
Services managed by the MAFC model and Cloud datacenters.

Figure 9 shows the simulation results. We divided the dataset randomly into 6 parts and we run these parts as shown in x axis. The y axis represents the number of services managed.

### 8.2. Energy Consumption

**Figure 10:**
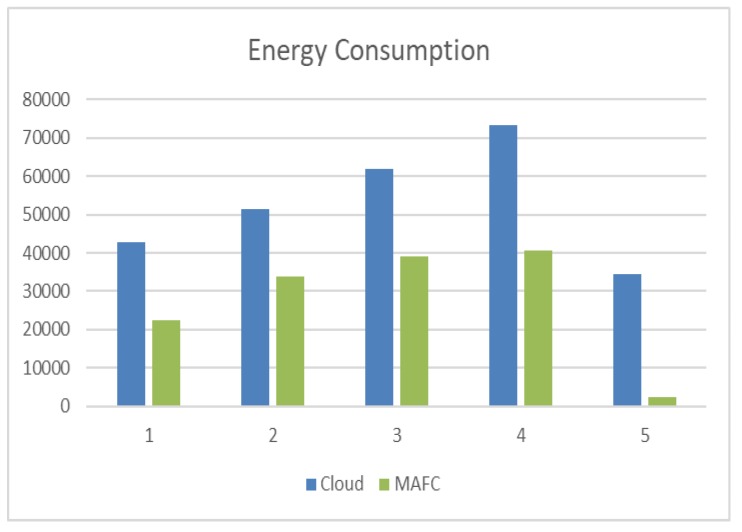
Energy consumption of MAFC and Cloud datacenters.

The simulation was targeting 5000 tasks with various number of PAs, in which when 4 PAs are connected to FNs, the consumed energy in FNs was 22,560 Joules and 42,781.4 Joules in Cloud datacenters. Whereas when 12 PAs are connected to FNs, the consumed energy in FNs was 33,840 Joules and 51,353 Joules in Cloud datacenters. Moreover, when the number of PAs increased to 14, the consumed energy in FNs was 39,120 Joules and 61,839.0 Joules in Cloud datacenters. Alternatively, when 18 PAs are connected to FNs, the consumed energy in FNs was 40,570 Joules and 73,305.4 Joules in Cloud datacenters. Lastly, when the number of PAs has been increased to 20, the consumed energy in FNs is 2355.56 Joules, and 34,590.3 Joules in Cloud datacenters. Figure 10 represents the results of this simulation. We divided the dataset randomly into five parts, and we run these parts.

### 8.3. Delay

**Figure 11:**
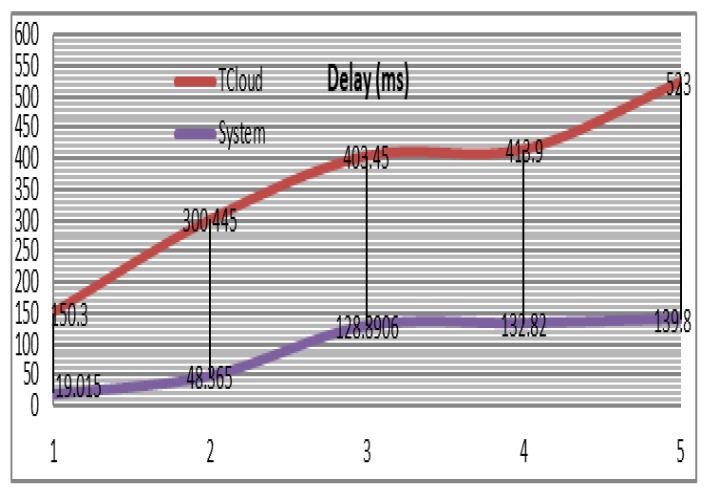
Delay factor in MAFC and Cloud datacenters.

The delay factor has been calculated in the simulation. An initial communication report five milliseconds delay from sensors to PAs has been assumed and the same value from PAs to FNs. A 105 milli second delay from FNs to Cloud has also assumed. In each iteration the initial delay is increasingly increased. The delay has been measured according to the lifecycle of the tasks, from the time of task received by personal agents until a processing completed. Sending tasks by multiple PAs in remote Cloud-based solution reduces the bandwidth segment, generates network congestion and higher round-trip time for results. As a result, the average network delay is high in the Cloud (Figure 11). In comparison, the average network delay in the Fog-based solution is low because there are numerous connections between the data source and the neighboring FNs.

The experimental evaluation shows the practicality of our proposed MAFC model for healthcare critical tasks management, where we simulate both the integrated architecture and the environment utilizing the iFogSim simulation toolbox. In two parts, we direct the simulation. From the start, the execution of the MAFC model for healthcare critical tasks management is compared with the Cloud-based solutions regarding energy usage, cost, and network delay considering computational assets are adequate in the Fog. After that, the service appropriation in Cloud-Fog integration is presented under various number of sensors and CPU use of the services while executing uses considering constrained computational assets in Fog. In the simulation protocol, the remaining synthetic task at hand is utilized, as presently the outstanding burden to recreate such conditions in a huge scope is not as of now accessible.

## 9. Conclusions

The Fog computing model MAS for managing critical healthcare application tasks was simulated that significantly manage Fog computing resources by providing two levels of task prioritization (locally and globally), scheduling, load balancing, and resource sharing through the involvement of MAS. The MAFC model provided efficient prioritization for abnormal tasks with efficient Tasks Scheduling for the patient critical situation and balanced network workload to local and global levels by estimating the cost of local and global workload. Lastly, it facilitated node cooperation and resource sharing with neighbor Nodes using Multi-Agents System. From experimental evaluation, the MAFC model is capable of process critical tasks locally or transferring to the appropriate resources. We follow a Java-created simulator (iFogSim). The real dataset is collected from the UTeM clinic and concerns data related to blood pressure and the critical patient’s calculation. In the scenario of this study, the decision to perform a task locally is assisted by a high priority, low load and high resource availability. The Services managed performance has been measured in terms of the number of services managed with the number of Cloud datacenters and the number of FNs. When the number of Cloud datacenters is two and the number of FNs is four, the number of tasks that are processed was 2546.2 in FNs, and 1797 tasks only have been processed in Cloud datacenters. Energy Consumption: the simulation targeted 5000 tasks with various numbers of PAs, in which when four PAs are connected to FNs, the consumed energy in FNs was 22,560 and 42,781.4 in Cloud datacenters. The delay factor has been calculated in the simulation. Five milliseconds is an initial communication delay from sensors to PAs that has been assumed and the same value from PAs to FNs. One hundred and five milliseconds has also assumed as a delay from FNs to Cloud. Nevertheless, the distributed decision-making process presented in this study has some limitations. The proposed method needs further experimental validation to test the system results using other dataset types such as ECG or EEG.

As future work, the authors plan to work on software improvements to install the simulator online. Moreover, it is planned to provide all the configuration settings, the dataset, and guidelines to reproduce the experiments. However, some considerations must be solved regarding the ethical committee permissions to conduct that procedure. The authors need to get the ethics approval from their institutions to guarantee the implementation of the right guidelines and standards to ensure anonymized data and the data privacy rules.

## Figures and Tables

**Table 1:** Dataset description.

Associated Tasks	Classification
Data Features	Multivariate
Attribute Characteristics	Real
Number of Attributes	3
Number of Instances	6740
Missing Values	0

**Table 2:** Normal, Moderate, and Critical blood pressure conditions.

Test Case	Conditions
1. Normal	90≤ Systolic ≤120
	60≤ Diastolic ≤80
2. Moderate	120≤ Systolic ≤140
	80≤ Diastolic ≤90
3. Critical	Systolic>140
	Systolic <90
	Diastolic <60
	Diastolic >90
